# Comprehensively characterize the soybean CAM/CML gene family, as it provides resistance against both the soybean mosaic virus and *Cercospora sojina* pathogens

**DOI:** 10.3389/fpls.2025.1633325

**Published:** 2025-07-21

**Authors:** Chunlei Zhang, Yanbo Wang, Ruiping Zhang, Rongqiang Yuan, Kezhen Zhao, Xiulin Liu, Xueyang Wang, Fengyi Zhang, Sobhi F. Lamlom, Bixian Zhang, Honglei Ren

**Affiliations:** ^1^ Soybean Research Institute of Heilongjiang Academy of Agriculture Sciences, Harbin, China; ^2^ College of Life Science, Northeast Agriculture University, Harbin, China; ^3^ Plant Production Department, Faculty of Agriculture Saba Basha, Alexandria University, Alexandria, Egypt; ^4^ Institute of Biotechnology of Heilongjiang Academy of Agricultural Sciences, Harbin, China

**Keywords:** soybean mosaic virus (SMV), *C. sojina*, soybean, CAM/CML genes, disease resistance

## Abstract

**Introduction:**

Calmodulin (CAM) and calmodulin-like (CML) proteins are essential calcium sensors that mediate plant responses to biotic and abiotic stresses. In soybean (Glycine max L. Merr.), these proteins play critical roles in resistance to multiple pathogens, yet a comprehensive characterization of this gene family and its connection to disease resistance has been lacking.

**Methods:**

This study identified and characterized 113 CAM/CML genes in the soybean genome, including 11 GmCAMs and 102 GmCMLs, through bioinformatic analysis using sequence homology, domain architecture, and phylogenetic approaches. Gene structure analysis, cis-acting element identification, and expression profiling were conducted to examine functional diversification and pathogen response patterns.

**Results:**

Phylogenetic analysis revealed 14 distinct groups with evidence of both ancient and recent gene duplication events contributing to family expansion. Gene structure analysis demonstrated higher conservation among GmCAMs (with all but one containing introns) compared to GmCMLs (70% intronless). Analysis of cis-acting elements indicated enrichment of hormone-responsive elements, particularly those associated with abscisic acid (31.2%) and methyl jasmonate (27.7%) responses. Expression profiling revealed distinct CAM/CML gene expression patterns in response to two major soybean pathogens: Soybean Mosaic Virus (SMV) and Cercospora sojina. We identified 15 GmCAM/CML genes that exhibited significantly altered expression in response to both pathogens, with GmCML23, GmCML47, and GmCAM4 showing the strongest correlation with resistance phenotypes.

**Discussion:**

The expression patterns of these genes were validated in various resistant and susceptible varieties, confirming their potential role in broad-spectrum disease resistance. Our findings offer valuable insights into the evolutionary history and functional diversification of the soybean CAM/CML gene family and identify promising candidates for enhancing soybean resistance to multiple pathogens through molecular breeding strategies.

## 1 Introduction

Plants encounter a variety of biotic and abiotic stresses throughout their growth and development, which can considerably diminish yields. They address these challenges through complex internal signaling pathways. Soybean plants (*Glycine max* L. Merr.) constantly encounter multiple challenges that can significantly affect growth, development, and yield (Majidian et al., 2024; Ren et al., 2024). As immobile organisms, soybeans are continually exposed to various environmental stimuli and pathogenic microorganisms, including bacteria, fungi, and viruses, all of which can lead to substantial crop losses (Ren et al., 2023). To survive these adverse conditions, soybeans have evolved sophisticated molecular mechanisms for perceiving external signals and translating them into appropriate cellular responses (Zhao et al., 2013).

Among the most economically significant pathogens affecting soybean production are Soybean Mosaic SMV and *C. sojina*, the causal agent of gray leaf spot (GLS) (Madhusudhan; Barro et al., 2023). SMV, a member of the Potyvirus genus, represents one of the most widespread and damaging viral diseases in soybean cultivation worldwide, causing yield losses ranging from 8% to 94% depending on cultivar susceptibility, infection timing, and environmental conditions (Choi et al., 2005; Zhang et al., 2009a). The virus is transmitted through aphid vectors and infected seeds, making it particularly challenging to control through conventional management practices. Similarly, *C. sojina* causes gray leaf spot, a foliar disease that has emerged as a major threat to soybean production, particularly in warm, humid environments (Kim et al., 2013). This fungal pathogen can cause premature defoliation, reduced photosynthetic capacity, and significant yield reductions of up to 60% in susceptible cultivars under favorable disease conditions (Yang and Luo, 2021). The increasing prevalence of both pathogens, combined with their potential for rapid spread and adaptation, underscores the critical need for understanding the molecular mechanisms underlying soybean resistance to these diseases (Jeena et al., 2024).

Calcium ions (Ca²^+^) serve as universal secondary messengers in these signaling networks, playing crucial roles in both regulating plant growth and mediating adaptations to biotic and abiotic stresses (Tong et al., 2021; Li et al., 2022). When environmental threats are detected, a rapid increase in cytoplasmic calcium concentration occurs, resulting in calcium transients and oscillations that represent the plant’s initial response to stimuli. Specialized calcium-binding proteins then decode these calcium signatures (Tuteja and Mahajan, 2007). In Arabidopsis, over 250 calcium sensor proteins have been identified, including calcineurin B-like proteins (*CBLs*), CaM, CMLs, calcium-dependent protein kinases (*CPKs*), and calcium and calmodulin-dependent protein kinase (*CCaMK*), and all of them contain different numbers of EF-hand motifs (Cheng et al., 2002; Day et al., 2002; Yang and Poovaiah, 2003; Luan, 2009; Boudsocq and Sheen, 2013; Wang et al., 2015).

CaMs/CMLs are essential types of Ca^2+^ sensors and are crucial components in Ca^2+^ signal transduction (Perochon et al., 2011). CaMs, which contain four EF-hand motifs, are conserved Ca^2+^ sensors found in both plants and animals (Mccormack and Braam, 2003). On the other hand, CMLs, which typically contain 1–6 EF-hand motifs, show some sequence similarity to CaM and display structural variations in plants (Gifford et al., 2007). Genome-wide identification and analysis of CaM/CML genes have been conducted for numerous plant species, including *Arabidopsis* (7 *CaMs* and 50 *CMLs*), rice (*Oryza sativa*, 5 *CaMs* and 32 *CMLs*), and *Brassica napus* (25 *CaMs* and 168 *CMLs*) (Yang and Poovaiah, 2003; Boonburapong and Buaboocha, 2007; He et al., 2020).

While CMLs and CaMs are homologous, plants have significantly larger CMLs than CaMs. The roles of CaMs and CMLs in stress response are well established. For instance, the AtCaM3 knockout mutant in Arabidopsis exhibits decreased heat tolerance, while transgenic lines overexpressing AtCaM3 demonstrate improved heat tolerance (Zhang et al., 2009b). *AtCML8* and *AtCML9* enhance Arabidopsis resistance to *Pseudomonas syringae* via the ABA and SA pathways (Zhu et al., 2017). The *AtCML24* gene plays a role in inhibiting pathogen-induced nitric oxide (NO) generation (Ma et al., 2008). In cotton, *GhCML11* interacts with *GhMYB108*, acting as a positive regulator in defense against *Verticillium dahliae* infection (Cheng et al., 2016). In the soybean genome, at least 262 genes encode proteins containing one to six EF-hand motifs, including 6 CaMs, 144 CMLs, 15 calcineurin B-like proteins, 50 calcium-dependent protein kinases (*CDPKs*), and various other calcium-responsive elements (Zeng et al., 2017; Ramlal et al., 2024). These proteins contain conserved EF-hand domains—helix-loop-helix structures that bind calcium and undergo conformational changes to activate downstream signaling pathways. CaM is evolutionarily conserved across eukaryotes and is the primary calcium sensor (Mohanta et al., 2017). Most soybean EF-hand genes (87.8%) contain at least one type of hormone signaling or stress response-related regulatory element in their promoter regions, indicating their potential involvement in stress adaptation mechanisms (Zeng et al., 2017). This extensive calcium-signaling network reflects the critical importance of these pathways in soybean’s ability to respond to the environment (Kaur et al., 2022).

Calcium signaling plays a significant role in soybean defense against pathogenic microorganisms (Negi et al., 2023). Specific calmodulin isoforms from soybean, such as *SCaM-4* and *SCaM-5*, are rapidly induced during plant defense responses and can enhance resistance to a wide spectrum of pathogens, including bacteria, fungi, and viruses (Ramlal et al., 2024). These specialized CaM variants appear to activate salicylic acid-independent pathways leading to disease resistance (Gao et al., 2014; Arfaoui et al., 2016). The soybean *GmCaM4* gene has been extensively studied in this context. Overexpression of *GmCaM-4* and *GmCaM-5*, two divergent calmodulin isoforms from soybean, can induce expression of pathogenesis-related (PR) genes and enhance disease resistance (Park et al., 2004). This activation depends on NIM1 (Non-immunity 1), a key regulator of systemic acquired resistance, demonstrating the integration of calcium signaling with established plant immune pathways (Park et al., 2020; Vidhyasekaran and Vidhyasekaran, 2020). Beyond pathogen defense, calcium signaling networks help soybeans adapt to abiotic stresses such as drought, salinity, and temperature extremes (Park et al., 2004). Osmotic stress induces a series of molecular and cellular responses beginning with increased cytosolic calcium concentration, which subsequently activates appropriate cellular mechanisms to mitigate potential damage (Ramlal et al., 2024).

For instance, the transcription factor *AtMYB2*, which regulates salt and dehydration-responsive genes, was identified as a calmodulin-binding protein (Zeng et al., 2015). The salt-inducible soybean CaM isoform *ScaM4* increases *AtMYB2’s* DNA binding activity, enhancing transcription of genes involved in proline synthesis and conferring salt tolerance (Ranty et al., 2006). This demonstrates how calcium signaling directly influences metabolic adaptations to environmental stress.

This study aims to thoroughly analyze the resistance mechanisms of the soybean disease resistance gene family to mosaic virus and *C. sojina*. By referencing published data on mosaic virus resistance and combining it with our own research on gray leaf spot, several significant gene loci related to disease resistance were identified, providing both theoretical foundations and practical guidance for breeding soybean disease resistance.

## 2 Materials and methods

### 2.1 Identification of CAM/CML genes in soybean

The soybean genome and GFF3 annotation files were obtained from the Soybase website. Seven *ATCAM* and fifty Arabidopsis *ATCML* proteins from the TAIR database were searched against protein sequences from the soybean genome database using BLASTP (E value < 1E-6). Additionally, hidden Markov model (HMM) profiles of the EF-hand domain (PF13499, PF13405, PF13202, PF13833) were downloaded from the PFAM database and utilized for HMM analysis with the TBtools tool. The results of BLASTP and HMM were compared and manually analyzed. The CAM/CML proteins were further evaluated using SMART (http://smart.embl-heidelberg.de/) and Interpro (http://www.ebi.ac.uk/interpro/), confirming the presence and integrity of the EF-hand domain without any other domains. The molecular weight (Mw) and isoelectric point (pI) of the *GmCAM/CML* proteins were predicted using the Protein Parameter Calc tool in TBtools.

### 2.2 Chromosome location, gene structure and conserved motif analysis

The chromosomal positions of CAM/CML genes were established by downloading the GFF file for the soybean genome and the CAM/CML family members from the Phytozome website using TBtools (Chen et al., 2023). Collinearity analysis of the CAM/CML genes was carried out with the one-step MCScanX plug-in of TBtools, and the results were visualized using the Advanced Circos plug-in (Chen et al., 2023). The Ka/Ks calculator in TBtools was utilized to compute nonsynonymous and synonymous substitution rates (Chen et al., 2023). The conserved *GmCAM/CML* protein motifs were predicted using the MEME program with default parameters, with a maximum of 5 motifs.

### 2.3 Multiple sequence alignment, phylogenetic analysis, and collinearity analysis

The Arabidopsis CAM/CML protein sequences (7 ATCAMS, 50 ATCMLS) were downloaded from the TAIR website. Multiple sequence alignments were conducted using the ClustalW tool. A rooted neighbor-joining phylogenetic (NJ) tree was constructed with Mega 11 software based on the full-length CAM/CML protein sequences to study the evolutionary relationships among CAM/CML proteins. The collinearity of CAM/CML genes was analyzed using the multiple scanning tool package MCSCANX, and the collinearity between homologous proteins in the Arabidopsis and rice genomes was examined. The results were visualized with TB-Tools. The KA/KS ratios among GmCAM/CML members were calculated using Ka/Ks_calculator2.0 software.

### 2.4 Prediction of cis-acting elements in the promoters of CAM/CML genes in soybean

We downloaded the promoter sequences (approximately 2 kb upstream of the transcription start sites) of soybean GmCAM/CML family genes from Phytozome, then used the PlantCARE online website (http://bioinformatics.psbugent.be/webtools/plantcare/html/) to predict and analyze promoter elements in these genes, remove the core elements (TATA-box and CAAT-box) of the promoter, and visualize them using TBtools (Chen et al., 2023).

### 2.5 Abiotic and biotic stress treatments

Five resistant soybean varieties (24JD21, 24JD210, 24JD829, 24JD890, 24JD697) and five susceptible (24JD892, 24JD878, 24JD876, 24JD882, 24JD867) were selected for biotic stress treatments based on their response to *C. sojina* infection. The *C. sojina* isolate (strain *CS-2018*) was obtained from the National Soybean Improvement Center in Nanjing, China, and cultured on potato dextrose agar (PDA) medium at 25°C for 14 days. Spores were harvested by washing the plates with sterile water containing 0.05% Tween-20, and the concentration was adjusted to 5 × 10^5^ spores/ml. Soybean plants were grown in a greenhouse under controlled conditions (25°C day/22°C night, 16-hour photoperiod, 70% relative humidity). Plants at the V3 stage (with the third trifoliate leaf fully developed) were used for inoculation. For *C. sojina* inoculation, the spore suspension was sprayed onto the plants until the runoff. The inoculated plants were kept in a humidity chamber for 48 hours and then returned to normal greenhouse conditions. Disease severity was assessed 14 days after inoculation using a 0–9 scale, where 0 = no lesions and 9 = severe infection with coalescing lesions covering more than 50% of the leaf area. Varieties with average scores of 0–3 were classified as resistant, 4–6 as moderately susceptible, and 7–9 as highly susceptible (**Supplementary Table S1**). For SMV response data, we utilized published transcriptome data from the comparative transcriptome analysis of early resistance of *Glycine max* to soybean mosaic virus (Li et al., 2023).

### 2.6 RNA extraction and RT-qPCR analyses

Total RNA was extracted using the RNA Extraction Kit from CWBIO (Jiangsu, China). This total RNA was reverse-transcribed to generate first-strand cDNA with the HiScript III RT SuperMix for qPCR (+gDNA wiper) provided by Vazyme (Beijing, China). Quantitative real-time PCR was conducted using a Roche instrument with ChamQ SYBR qPCR Master Mix from Vazyme (Beijing, China). The internal reference gene utilized was soybean β-Tubulin, and the relative expression levels of the target gene were calculated with the 2^-ΔΔCT method. In the expression profile analysis, significance was evaluated via the Student t-test, with levels of significance indicated by asterisks (*P < 0.05, **P < 0.01, and *** P < 0.001). The sequences of primers used in this study are listed in **Supplementary Table S2**. For the *C. sojina* infection time course, leaf samples were gathered 14 days after inoculation. Each variety had three biological replicates, with each replicate comprising the third trifoliate leaves from three plants. The collected leaf tissues were promptly frozen in liquid nitrogen and stored at -80°C until RNA extraction.

## 3 Results

### 3.1 Identification and annotation of CAM/CML gene family members in soybean

To identify CAM/CML genes in soybean, we employed a dual screening approach. First, we conducted BLASTP searches against the soybean genome using protein sequences of seven *ATCAMs* and fifty *ATCMLs* from Arabidopsis as queries, with an e-value threshold of 1 × 10^-6. Concurrently, we performed hidden Markov model (HMM) analyses by aligning AMP domain files (PF13499, PF13405, PF13202, PF13833) with all soybean amino acid sequences. Candidate genes identified through these methods were further verified using multiple domain databases including PFAM, SMART, and NCBI’s Conserved Domain Database (CDD). This comprehensive screening approach resulted in the identification of 11 *GmCAM* and 102 *GmCML* genes. These genes were systematically named according to their chromosomal positions (*GmCAM1-GmCAM11* and *GmCML1-GmCML102*). Subsequent characterization of the physical and chemical properties of these CAM/CML family members revealed considerable diversity. The encoded proteins ranged in length from 74 to 371 amino acids, with theoretical molecular weights between 8622.56 and 43574.75 kDa. The isoelectric points (pI) of these proteins exhibited a broad distribution from 3.94 to 9.99, indicating diverse charge properties within this gene family. Detailed information for individual family members is provided in **Supplementary Table S3**.

### 3.2 Phylogenetic relationships reveal distinct evolutionary groups within the CAM/CML gene family

**Figure 1:**
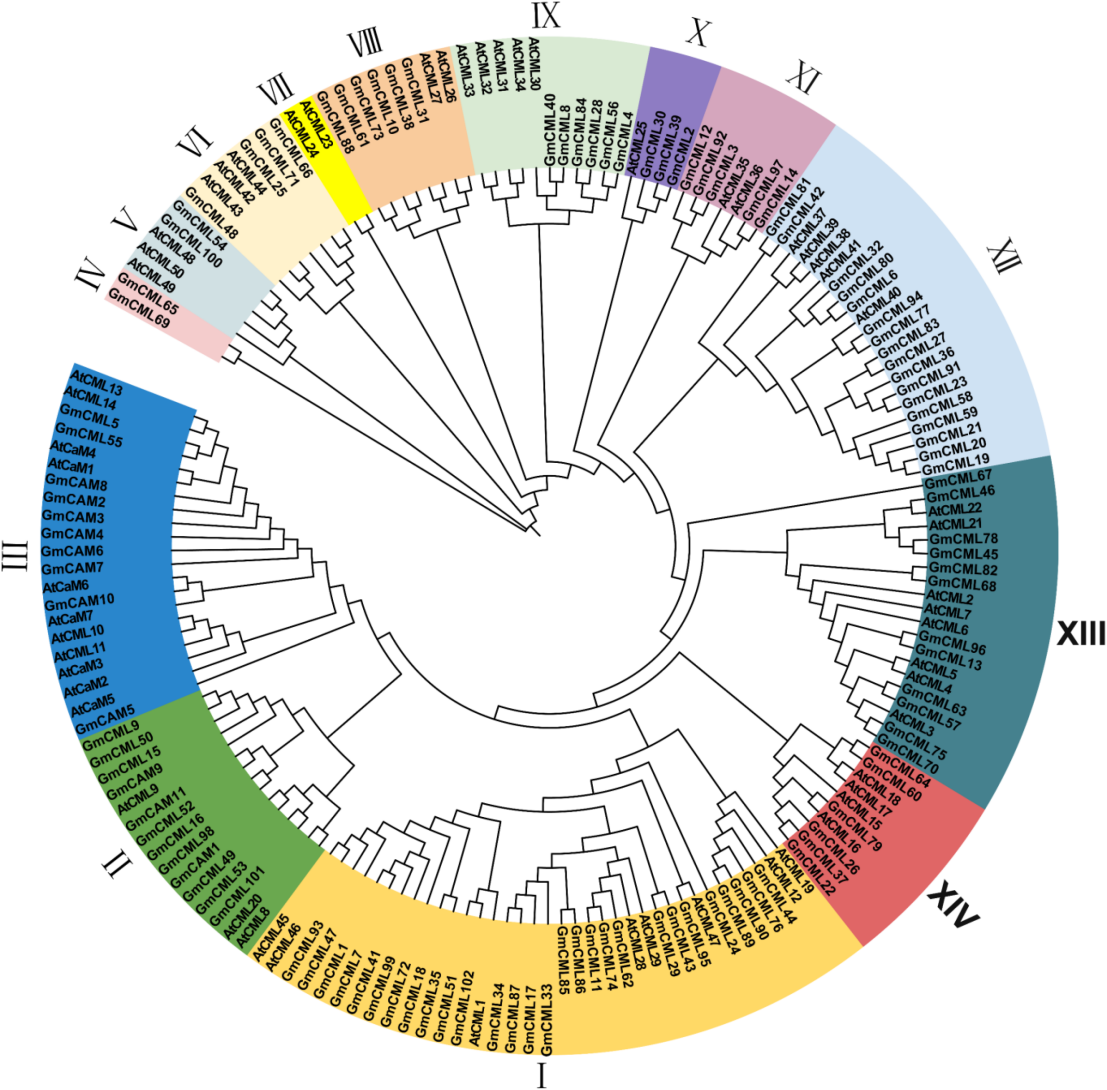
Phylogenetic tree of CAM/CML gene family members. Phylogenetic trees were built using MEGA-X software following the neighbor-joining (NJ) method and 1000 bootstrap replications were performed. Visualizing evolutionary trees using EvolView. All CAM/CML members are divided into 14 groups and presented in different colors.

To investigate the evolutionary relationships among CAM/CML proteins, we constructed a neighbor-joining phylogenetic tree using maximum likelihood methods with 1,000 bootstrap replicates. The analysis included 57 Arabidopsis proteins (7 *AtCAMs* and 50 *AtCMLs*) and 113 soybean proteins (11 GmCAMs and 102 GmCMLs) (**Figure 1**). The phylogenetic analysis resolved the CAM/CML superfamily into 14 distinct monophyletic groups with strong bootstrap support (>70%). Group I emerged as the largest clade, containing 36 members (8 *AtCMLs* and 28 *GmCMLs*), suggesting an ancient expansion of CML proteins predating the Arabidopsis-soybean divergence approximately 54 million years ago. Groups II and III collectively harbored all CAM proteins, with Group II containing 15 members (3 *AtCMLs*, 9 *GmCMLs*, and 3 *GmCAMs*) and Group III containing 21 members (4 *AtCMLs*, 7 *AtCAMs*, 8 *GmCAMs*, and 2 *GmCMLs*). The exclusive clustering of CAM proteins within these two groups indicates their evolutionary origin from a common ancestral lineage and subsequent conservation of core calcium-sensing functions. The remaining groups (IV-XIV) comprised exclusively CML proteins, with variable sizes ranging from 2 to 22 members. Notably, Groups IV and VII represented the smallest clades, each containing only 2 members. Group IV exclusively comprises *GmCML* proteins (*GmCML*_65 and *GmCML*_69), while Group VII contained only *AtCML* proteins (*AtCML*_23 and *AtCML*_24). This species-specific clustering pattern suggests recent gene duplication events following the Arabidopsis-soybean divergence, potentially indicating lineage-specific functional adaptations. The phylogenetic distribution reveals several key evolutionary patterns. First, the extensive diversification of CML proteins (152 total) compared to CAM proteins (18 total) demonstrates significant sub functionalization following ancient duplication events. Second, the mixed species composition in most groups indicates that major gene family expansions occurred before monocot-dicot divergence. Third, the isolated positioning of the *GmCML* pair in Group IV, separated by long branch lengths from other clades, suggests potential neofunctionalization and acquisition of specialized calcium-sensing roles unique to legumes. Bootstrap values exceeding 85% for major nodes confirm the robustness of these phylogenetic relationships. The clear separation between CAM and CML lineages, combined with species-specific clustering patterns in smaller groups, provides a framework for understanding the evolutionary forces shaping calcium signaling diversity in flowering plants.

### 3.3 Conserved motif and gene structure analysis of CAM/CML genes

**Figure 2:**
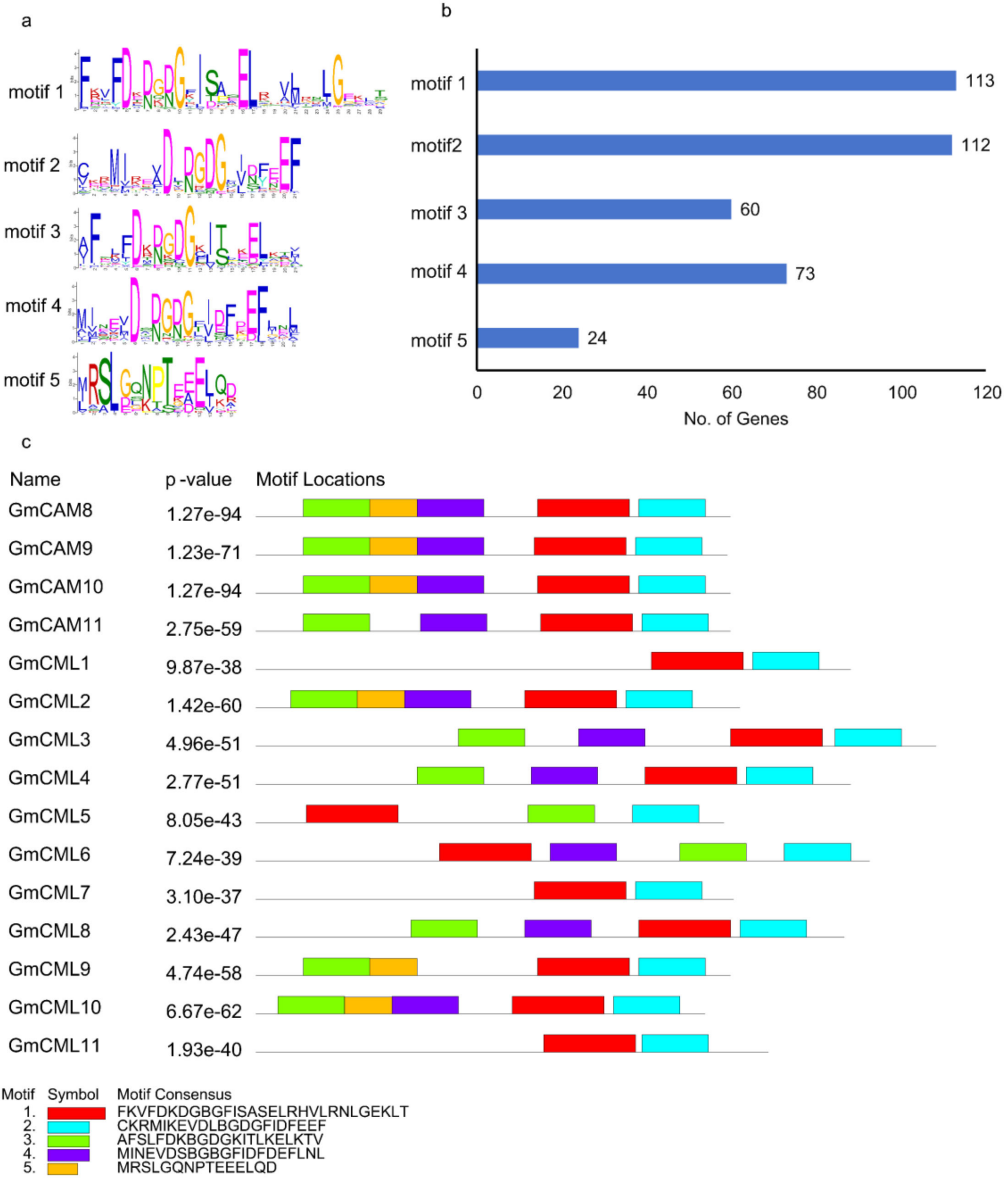
Conserved motif identification and structural organization of soybean CAM/CML proteins. **(a)** Position weight matrices showing amino acid conservation patterns for the five identified motifs. Letter height represents the relative frequency and information content at each position. **(b)** Distribution frequency of motifs across the CAM/CML gene family showing the number of genes containing each motif. **(c)** Motif architecture and genomic organization of 15 CAM/CML genes with statistical significance values (p-values). Colored blocks represent different motifs within each protein sequence.

A comprehensive motif analysis of soybean CAM/CML genes was performed using the MEME suite to elucidate conserved structural and functional domains within this important calcium-binding protein family. Position weight matrix analysis revealed distinct amino acid conservation patterns within each motif (**Figure 2a**). The analysis identified five distinct conserved motifs across 15 CAM/CML genes from the Williams 82 reference genome (Wm82.gnm4.ann1), with statistical significance values ranging from 1.93 × 10^-40 to 1.27 × 10^-94 (**Figure 2c**). Motif distribution analysis revealed differential patterns of conservation across the gene family (**Figure 2b**). Motifs 1 and 2 demonstrated near-universal presence, occurring in 113 and 112 genes respectively, indicating their fundamental importance in CAM/CML protein structure and function. Motif 4 was present in 73 genes, while Motif 3 occurred in 60 genes. Notably, Motif 5 showed the most restricted distribution, appearing in only 24 genes, suggesting a specialized functional role within a subset of the CAM/CML family. Analysis of motif combinations revealed a modular architecture pattern across the CAM/CML gene family (**Figure 2c**). Individual proteins contained between one and five motifs in various combinations, reflecting the evolutionary plasticity of this gene family. The most statistically significant proteins, including *GmCML 8*, *GmCML10* (both p = 1.27 × 10^-94), and *GmCML9* (p = 1.23 × 10^-71), displayed complex motif arrangements with multiple domain repeats. Several distinct architectural patterns emerged: (1) proteins containing primarily Motifs 1 and 2, representing the core CAM/CML structure; (2) proteins with additional Motifs 3 and/or 4, suggesting expanded functional capacity; and (3) a specialized subset containing Motif 5, potentially representing functionally divergent family members. This modular organization indicates that CAM/CML proteins have evolved through domain duplication and recombination events, allowing for functional diversification while maintaining essential calcium-binding capabilities.

The exceptionally high statistical significance of motif conservation (all p-values < 10^-30) demonstrates strong evolutionary pressure to maintain these structural elements, consistent with their critical role in calcium-mediated cellular processes. The universal presence of Motifs 1 and 2 across nearly all analyzed genes indicates these represent the essential functional core of CAM/CML proteins, likely corresponding to canonical EF-hand calcium-binding domains. The restricted distribution of Motif 5 to a subset of genes suggests functional sub familiarization within the CAM/CML family. Proteins containing this motif may possess specialized roles in specific calcium signaling pathways or exhibit distinct regulatory mechanisms. The intermediate distribution patterns of Motifs 3 and 4 indicate additional layers of functional complexity, potentially conferring differential calcium sensitivity, protein-protein interaction capabilities, or subcellular localization signals. The identification of 15 CAM/CML genes with conserved motifs across multiple chromosomes indicates significant gene family expansion in soybean. The modular nature of motif combinations suggests that this expansion occurred through various mechanisms including tandem duplication, segmental duplication, and subsequent domain shuffling events.

### 3.4 Gene structure organization and domain architecture of CAM/CML family members

**Figure 3:**
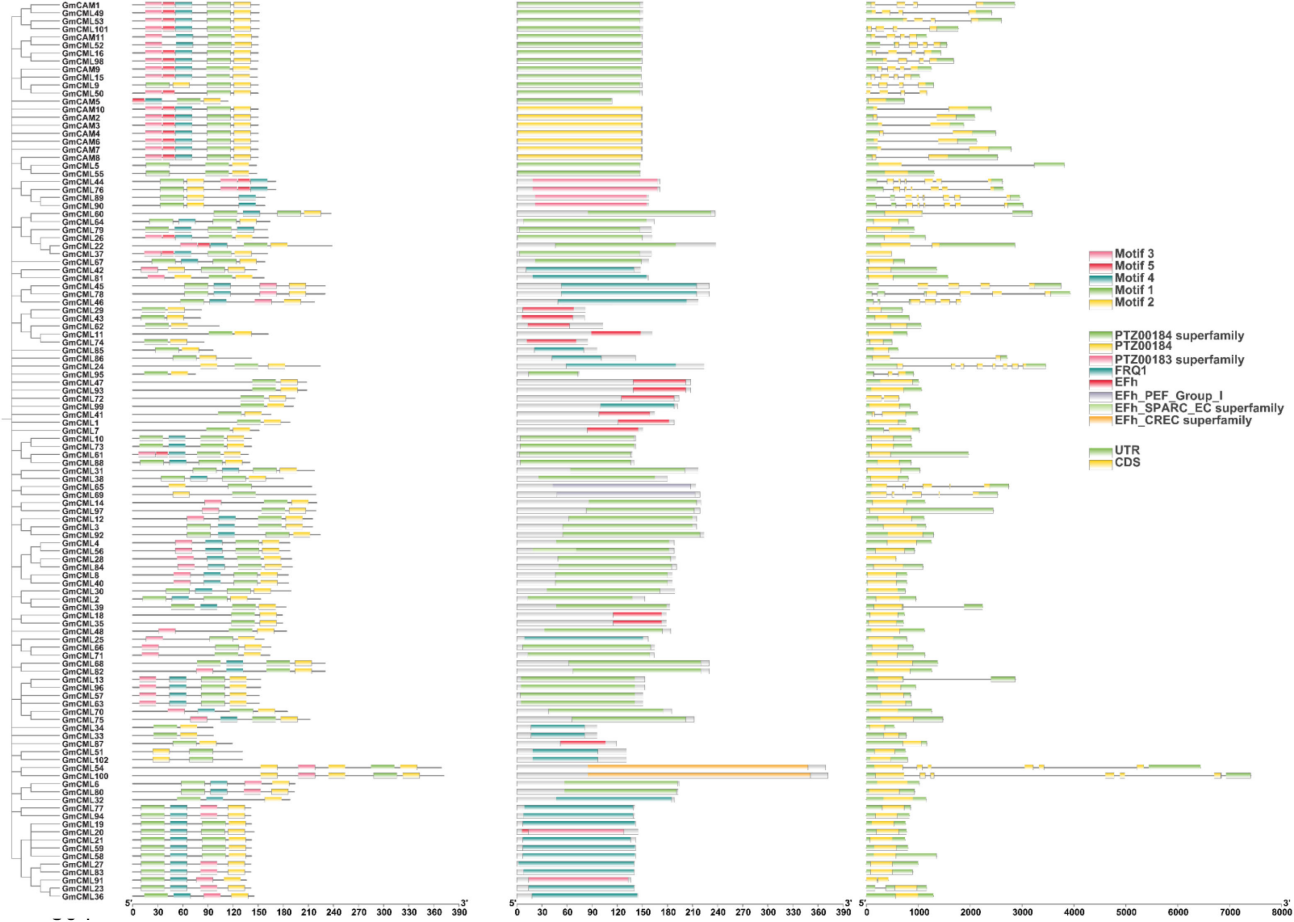
Gene structure organization and domain architecture of soybean CAM/CML family members. Schematic representation showing the complete gene structures of 11 *GmCAM* and 102 *GmCML* genes, including coding sequences (CDS), untranslated regions (UTR), and conserved protein domains. Motifs 1–5 identified through MEME analysis are mapped to their genomic locations. Protein domains include EF-hand domains (EFh) and their subtypes, PTZ superfamily domains, and FRQ1 domains. Scale bars indicate nucleotide positions. The organization demonstrates the modular architecture and evolutionary diversification of the CAM/CML gene family, with CAM genes showing more uniform structures while CML genes display remarkable structural diversity.

Gene structure analysis of the soybean CAM/CML family revealed distinct organizational patterns across 11 *GmCAM* and 102 *GmCML* genes (**Figure 3**). CAM genes exhibited relatively uniform structures with compact organization, while CML genes showed remarkable diversity, ranging from simple architectures to complex, multi-domain organizations extending over 8,000 base pairs. The five conserved motifs identified through MEME analysis were mapped to their genomic locations within gene structures. Motifs 1 and 2 were consistently located within coding sequences across all genes, confirming their essential functional roles. Motif 5 was predominantly found in CAM subfamily members, supporting functional specialization between CAM/CML subfamilies. Domain annotation revealed multiple EF-hand domain subtypes across the family, including *EFh_PEF*_Group_I, *EFh_SPARC_EC* superfamily, and *EFh_CREC* superfamily domains (**Figure 3**). *PTZ00184* and *PTZ00183* superfamily domains were identified in multiple members, suggesting additional functional capabilities beyond calcium binding. The FRQ1 domain was detected in a subset of genes, potentially conferring specialized regulatory functions. Most genes contained multiple EF-hand domains arranged in tandem, consistent with the typical architecture of calcium-binding proteins, which require multiple coordination sites. The modular arrangement supports evolution through domain duplication and recombination mechanisms. CAM genes demonstrate uniform structures with consistent domain arrangements, reflecting their conserved role as primary calcium sensors. The specific presence of Motif 5 in CAM genes provides a molecular basis for their unique regulatory properties. CML genes exhibited greater structural diversity with varying EF-hand domain combinations and accessory motifs, correlating with their functional specialization for distinct cellular processes. The structural diversity observed across the CAM/CML family demonstrates the evolutionary mechanisms that have shaped functional diversification, with CAM genes under stronger purifying selection while CML genes have undergone extensive structural innovation to generate the current functional versatility of plant calcium signaling networks.

### 3.5 Chromosomal distribution and gene structure analysis of *GmCAMS/CML*

**Figure 4:**
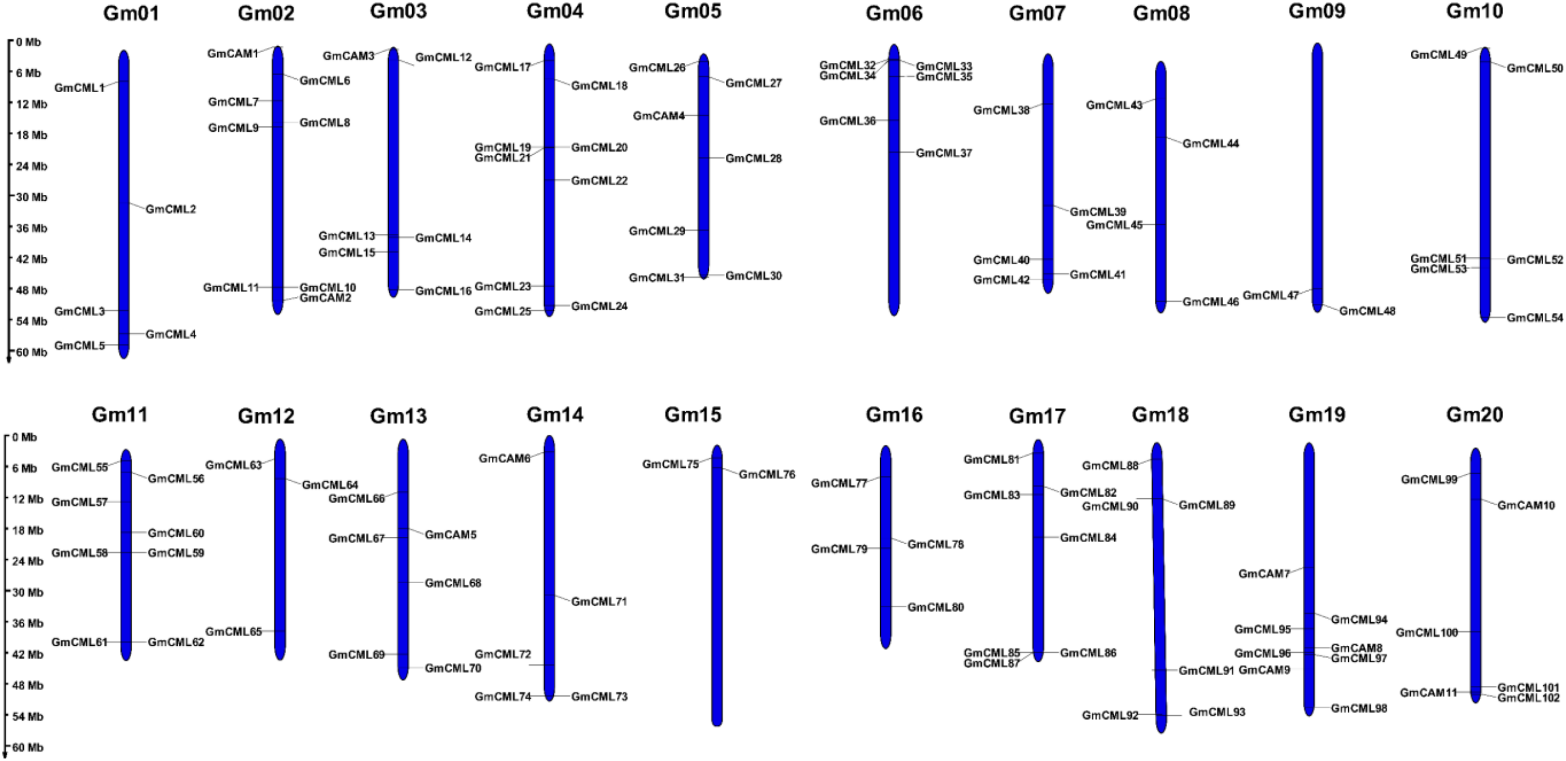
Chromosomal distribution of calmodulin (CAM) and calmodulin-like (CML) genes in the soybean genome. The chromosomal locations of 11 *GmCAM* and 102 *GmCML* genes were mapped across the 20 soybean chromosomes (Gm01-Gm20). Yellow bars represent chromosomes with the relative positions of genes indicated in megabases (Mb) from top (0 Mb) to bottom (60 Mb). Gene names are displayed adjacent to their respective positions. Note the uneven distribution pattern, with some chromosomes harboring multiple genes while others contain fewer. Clusters of genes in proximity suggest tandem duplication events, while the widespread distribution across all chromosomes reflects the importance of calcium signaling throughout the soybean genome.

A genome-wide analysis revealed the presence of 11 CAM and 102 CML genes distributed across all 20 chromosomes of the soybean genome. The distribution pattern exhibited significant variation, with chromosomes Gm11, Gm17, and Gm04 containing the highest number of CML genes, while the CAM genes were confined to only 7 chromosomes (Gm02, Gm03, Gm05, Gm13, Gm14, Gm19, and Gm20). Notably, we identified multiple clustering patterns of CML genes in specific chromosomal regions, suggesting potential tandem duplication events during evolution. The substantial expansion of the CML gene family (102 members) compared to the more conserved CAM family (11 members) suggests functional diversification following the whole-genome duplication events known to have occurred in soybean. This comprehensive chromosomal mapping provides important insights into the evolutionary history and potential functional specialization of calcium-signaling genes in Glycine max (**Figure 4**).

### 3.6 Cis-acting element analysis

**Figure 5:**
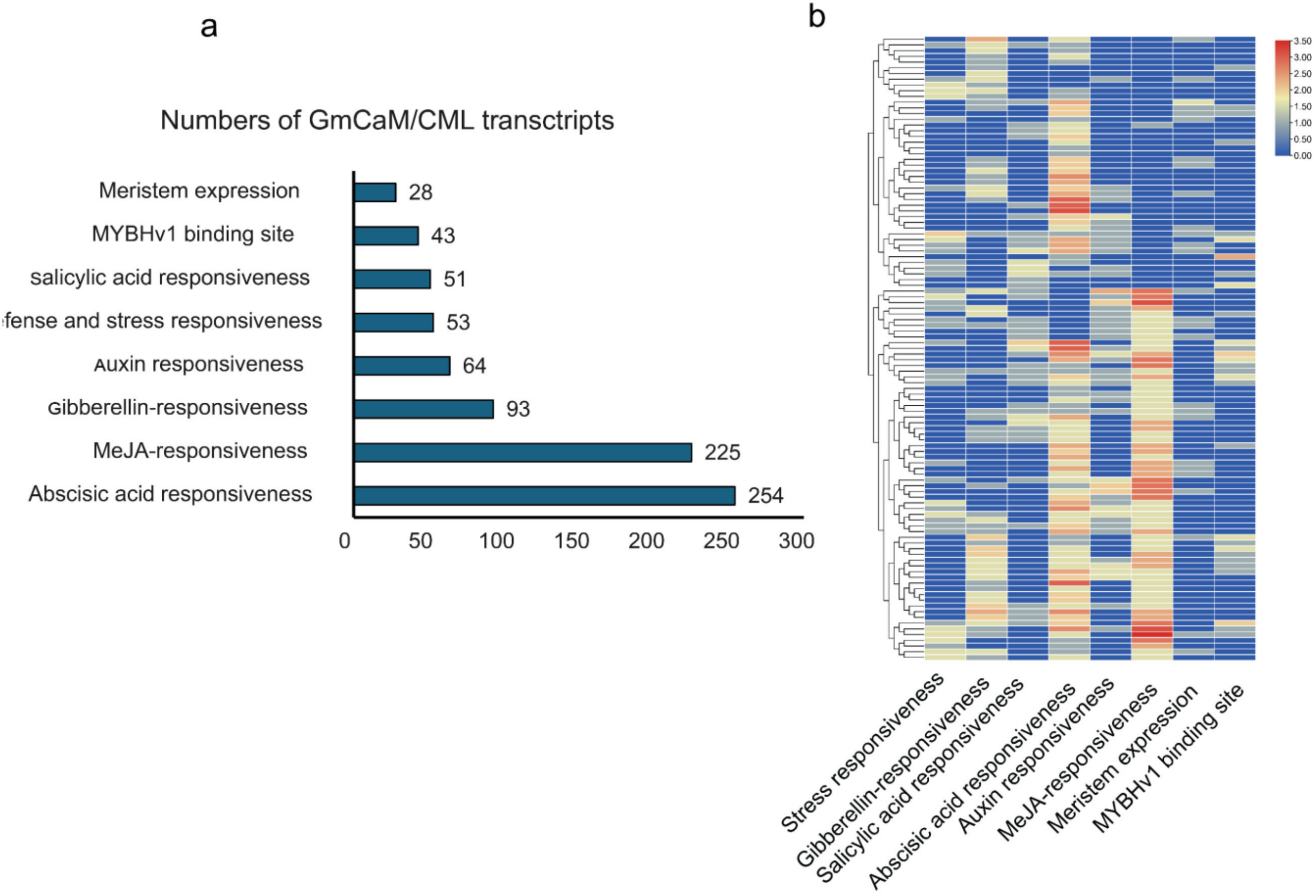
Cis-regulatory element analysis of *GmCaM/CML* genes. **(a)** Distribution of cis-acting elements in the 2 kb upstream regions of *GmCaM/CML* genes, categorized by function. **(b)** Hierarchical clustering heatmap showing the distribution pattern of different cis-regulatory elements across *GmCaM/CML* family members. Color intensity represents the abundance of each element type, with red indicating higher abundance.

Comprehensive analysis of the 2 kb upstream sequences of *GmCaM/CML* genes revealed a striking enrichment of hormone-responsive cis-acting elements (**Figure 5a**). Abscisic acid (ABA) responsive elements constituted the largest proportion (254 elements, 31.2%), followed closely by methyl jasmonate (MeJA) responsive elements (225 elements, 27.7%). Gibberellin-responsive elements (93 elements, 11.4%) and auxin-responsive elements (64 elements, 7.9%) were also well-represented. Elements associated with biotic and abiotic stress responses comprised a notable fraction, with defense/stress-responsive elements and salicylic acid-responsive elements accounting for 6.5% and 6.3%, respectively. *MYBHv1* binding sites (5.3%) and meristem expression elements (3.4%) were less abundant but present across multiple genes. Hierarchical clustering analysis revealed distinct cis-regulatory patterns among *GmCaM/CML* family members (**Figure 5b**), suggesting potential functional specialization. Several genes exhibited strong enrichment for specific hormone-responsive elements, while others displayed more diverse regulatory signatures. This regulatory diversity likely underpins the multifaceted roles of calmodulin and calmodulin-like proteins in calcium-mediated signaling networks, particularly in hormone signaling pathways and stress responses. The prevalence of hormone-responsive elements highlights the pivotal role of *GmCaM/CML* proteins as integration nodes between calcium signaling and hormone-regulated processes in soybean.

### 3.7 Extensive gene duplication and evolutionary conservation underpin *GmCaM/CML* gene family expansion

**Figure 6:**
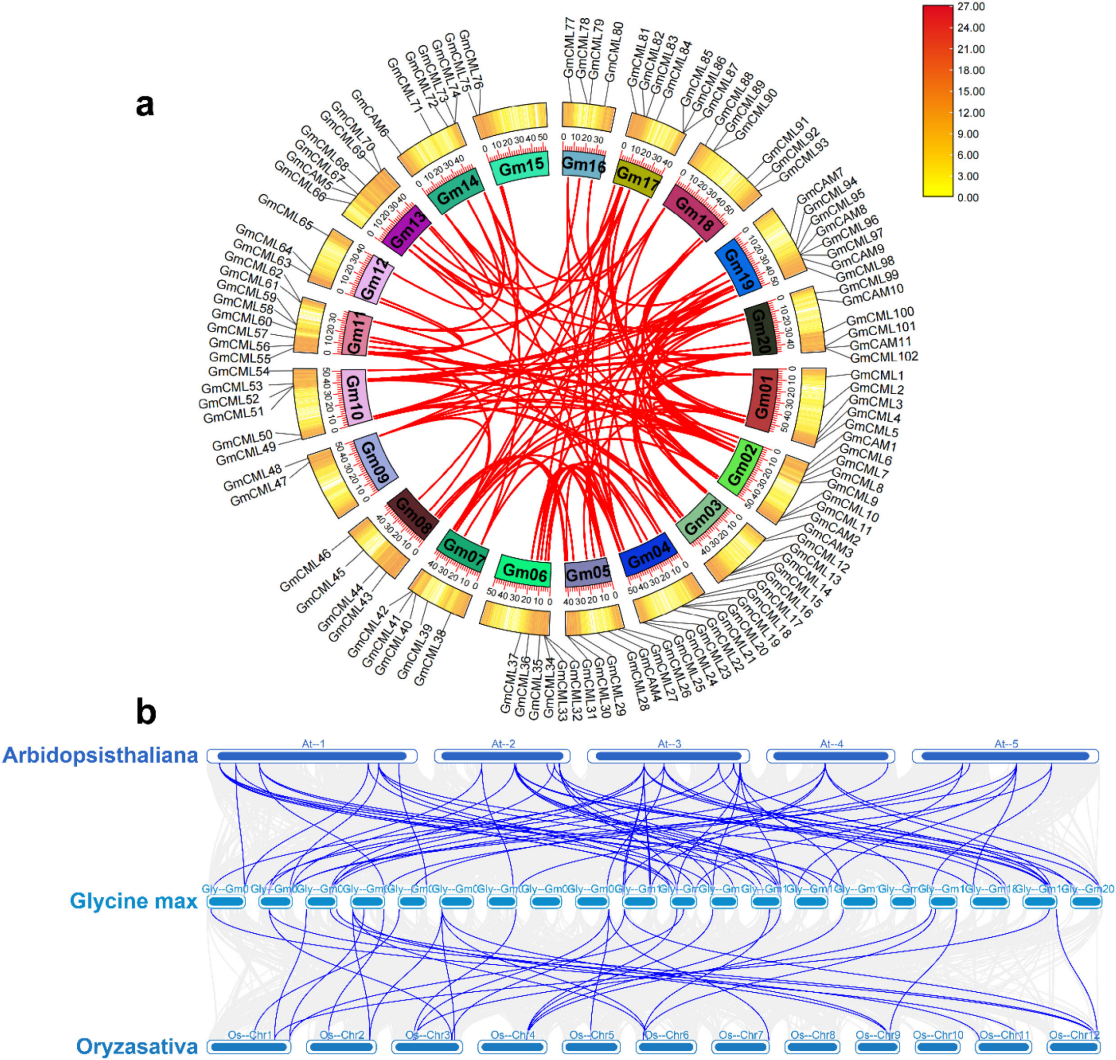
Collinearity analysis reveals duplication patterns and evolutionary conservation of the CaM/CML gene family. **(a)** Circular diagram showing homologous relationships (red lines) among 117 pairs of GmCaM/CML genes across soybean chromosomes. Colored blocks represent different chromosomes with gene distributions. Colored blocks represent different chromosomes with gene distributions. The color scale (0.00–27.00) indicates gene density per chromosomal region, where yellow represents low gene density and dark red represents high gene density. **(b)** Syntenic relationships of CaM/CML genes between Arabidopsis thaliana (top), Glycine max (middle), and Oryza sativa (bottom). Blue lines connect homologous genes across species, illustrating patterns of evolutionary conservation and divergence.

Collinearity analysis revealed substantial duplication events contributing to the expansion of the *CaM/CML* gene family in soybean (**Figure 6a**). We identified 117 pairs of homologous *GmCaM/CML* genes distributed across the soybean genome, visualized as connecting lines in the circular diagram. This extensive duplication pattern likely resulted from the two whole-genome duplication events in soybean’s evolutionary history. Comparative genomic analysis between soybean and model plant species uncovered significant evolutionary relationships in the *CaM/CML* gene family (**Figure 6b**). We identified 67 pairs of homologous CaM/CML genes between soybean and Arabidopsis, and 32 pairs between soybean and rice. This differential homology pattern reflects the closer evolutionary relationship between soybean and Arabidopsis (both dicots) compared to rice (monocot). To assess evolutionary selection pressure, we calculated Ka/Ks ratios for all homologous gene pairs. Notably, all gene pairs exhibited Ka/Ks ratios below 1.0, indicating strong purifying selection (**Supplementary Table S4**). This evolutionary constraint suggests functional conservation of CaM/CML proteins across species, highlighting their fundamental importance in calcium-mediated signaling processes throughout plant evolution. The greater number of homologous relationships between soybean and Arabidopsis compared to rice aligns with established phylogenetic relationships between these species and suggests lineage-specific expansion and conservation patterns in the plant CaM/CML gene family.

### 3.8 Tissue-specific expression reveals functional diversification of *GmCaM/CML* genes

**Figure 7:**
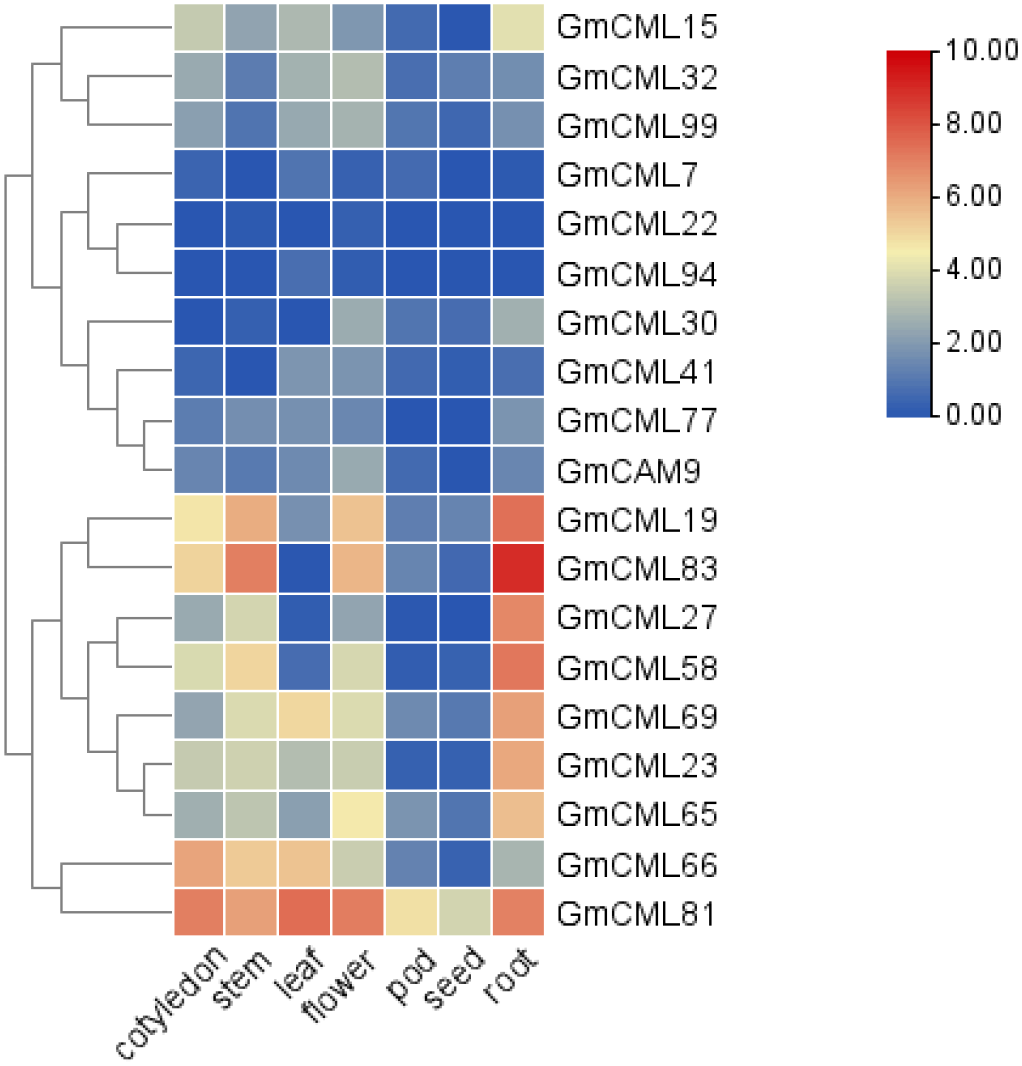
Tissue-specific expression profiles of GmCaM/CML genes. Heatmap showing expression patterns of selected GmCaM/CML genes across seven soybean tissues (cotyledon, stem, leaf, flower, pod, seed, and root) based on RNA-Seq data. Hierarchical clustering reveals groups with similar expression patterns. Color scale indicates normalized expression levels, with blue representing low expression and red representing high expression (scale 0-10).

Analysis of RNA-Seq data across soybean tissues revealed distinct spatial expression patterns among *GmCaM/CML* family members (**Supplementary Figure S1**). Hierarchical clustering identified two major expression clusters: one predominantly showing low expression across most tissues (upper cluster including *GmCML15* through *GmCAM9*), and another exhibiting moderate to high expression in various tissue combinations (lower cluster including *GmCML19* through *GmCML81*) (**Figure 7**). Several genes displayed striking tissue-specific expression profiles. *GmCML81* demonstrated robust expression across vegetative tissues but minimal activity in seeds. *GmCML83* showed high expression across multiple tissues, particularly in roots. Conversely, *GmCML22* exhibited preferential expression in flowers and roots, while *GmCML94* showed enhanced expression in leaves and flowers, suggesting specialized functions in these tissues. Notably, while phylogenetically related genes often shared similar expression patterns, we observed several instances of expression divergence among duplicated genes. For example, *GmCML83* maintained high expression across all tissues examined, whereas its homolog *GmCML94* was expressed at substantially lower levels. This expression divergence following gene duplication suggests neo- or sub-functionalization events contributing to the functional diversification of the *GmCaM/CML* gene family in soybean.

### 3.9 Differential expression profiles of 19 calmodulin-like genes in resistant and susceptible soybean varieties in response to *C. sojina* infection

**Figure 8:**
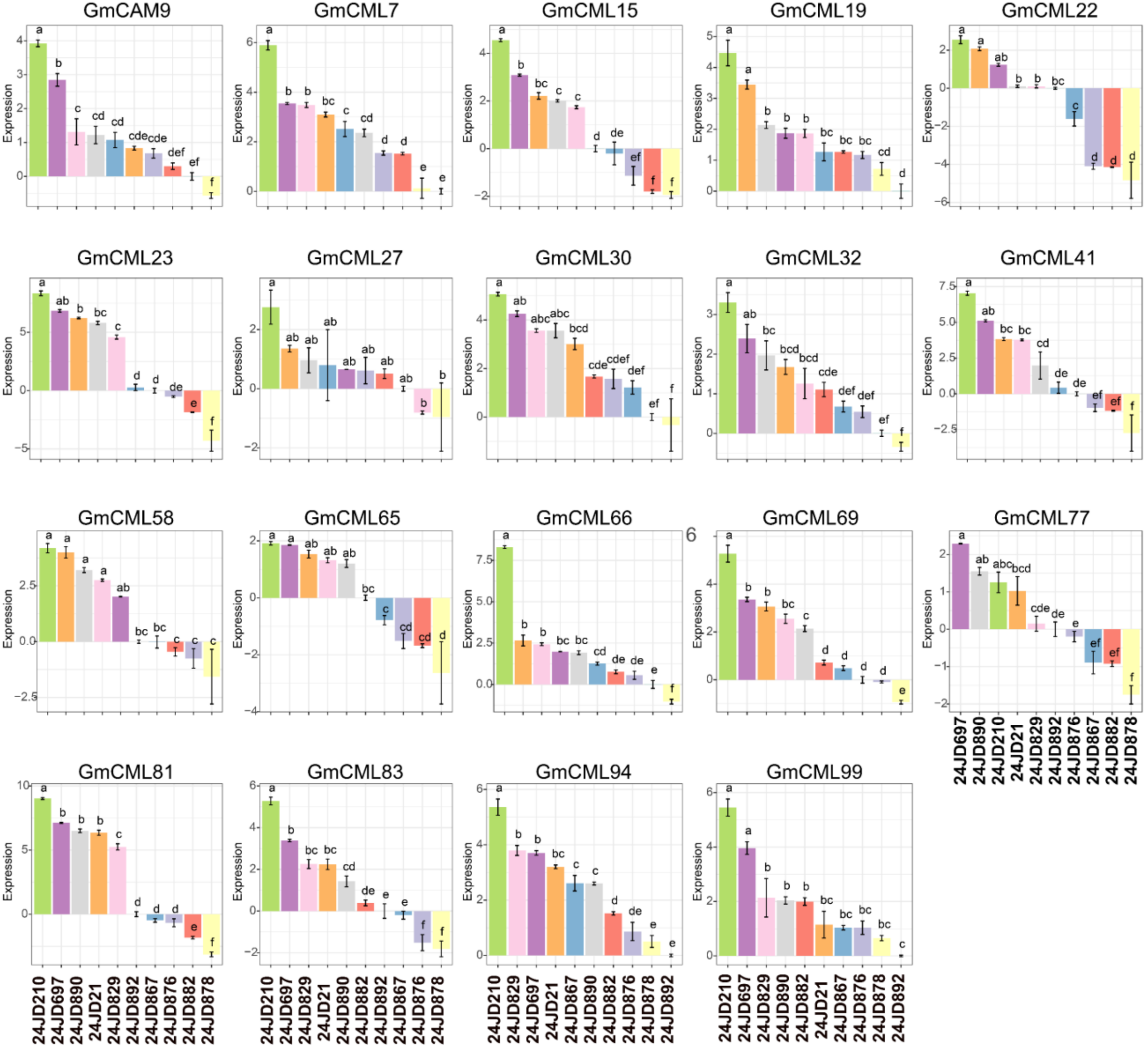
Differential expression profiles of 19 calmodulin-like genes in resistant and susceptible soybean varieties in response to *C. sojina* infection.

Transcriptome analysis of 19 CML genes in Glycine max revealed distinct expression patterns correlated with resistance to *C. sojina* infection (**Figure 8**). Expression profiles clustered into three categories across the five resistant and five susceptible varieties: high-magnitude (GmCML32, GmCML65, GmCML77), moderate (GmCML7, GmCML15, GmCML66, GmCML83, GmCML94), and low-variable (GmCML19, GmCML22, GmCML23, GmCML41, GmCML58, GmCML69). GmCML77 exhibited the highest expression amplitude (10 relative units) in resistant varieties, particularly in samples 24JD210, 24JD697, and 24JD890. Interestingly, GmCML32 and GmCML65 exhibited substantial dynamic ranges (10 and 7.5 units, respectively), with significant upregulation in resistant varieties following infection. Conversely, *GmCML23* maintained a relatively stable expression (± 2 units) regardless of the resistance phenotype, suggesting a constitutive rather than an inducible role. Temporal expression patterns following *C. sojina* challenge fell into three categories: progressive increase (*GmCML15, GmCML32*), bell-shaped (*GmCAM9, GmCML27, GmCML30*), and oscillatory (*GmCML22, GmCML69, GmCML77*). The resistant varieties showed distinctive early upregulation of *GmCML94 and GmCML99*, while late-stage infection was characterized by elevated expression of *GmCML7, GmCML32*, and *GmCML65* in these same varieties (**Figure 8**). Five genes (*GmCML19*, *GmCML22, GmCML41, GmCML58, GmCML69*) demonstrated negative expression values in susceptible varieties relative to resistant ones, indicating potential suppression during pathogen infection. This was most pronounced in *GmCML22*, which exhibited the widest expression range (-5 to +5 units) between resistant and susceptible varieties. Notably, these repression patterns were primarily associated with early infection stages in susceptible varieties, suggesting pathogen-induced transcriptional interference. Sample-specific expression analysis revealed differential responses among resistant varieties (24JD210, 24JD697, and 24JD890) compared to susceptible varieties (24JD829, 24JD882, and 24JD892) (**Figure 8**). This divergence suggests functional specialization among CML family members may contribute to *C. sojina* resistance. Particularly, the coordinated expression of *GmCML77, GmCML81, and GmCML83* in resistant varieties points to a potential calcium-signaling cascade essential for mounting effective defense responses against this economically significant pathogen.

## 4 Discussion

In this study, we conducted a genome-wide identification of *GmCaM* and GmCML genes in soybean, leading to the discovery of 11 *GmCAMs* and 102 *GmCMLs*, along with their collinearity, structures, chromosomal locations, and expression patterns across various tissues. Additionally, we analyzed the differential expression profiles of *GmCaM* and *GmCML* genes in response to SMV and *C. sojina* infection. This study offers comprehensive insights into soybean’s *GmCaM* and *GmCML* gene families.

### 4.1 Evolutionary insights and family expansion of soybean CAM/CML genes

Our comprehensive analysis identified 113 CAM/CML genes (11 *GmCAMs* and 102 *GmCMLs*) in the soybean genome, representing one of the most prominent CAM/CML families reported in plants. This extensive gene family size compared to Arabidopsis (7 *AtCAMs* and 50 *AtCMLs*) (Mccormack et al., 2005), and rice (5 *OsCAMs* and 32 *OsCMLs*) (Boonburapong and Buaboocha, 2007), likely reflects the paleopolyploid nature of the soybean genome, which has undergone two whole-genome duplication events approximately 59 and 13 million years ago (Schmutz et al., 2010). The collinearity analysis revealed that most *GmCAM/CML* genes (117 pairs) resulted from these duplication events, significantly contributing to family expansion. The phylogenetic analysis revealed 14 distinct groups of varying sizes and compositions, indicating different gene retention rates and loss following duplication events. The presence of mixed species composition in most groups suggests that significant diversification of the CAM/CML family occurred before the divergence of monocots and dicots, estimated at 140–150 million years ago (Chaw et al., 2004; Liu et al., 2024). However, the species-specific clustering observed in some groups (e.g., Group IV, which contains only *GmCMLs*) suggests more recent lineage-specific duplication and potential neofunctionalization. The extremely low Ka/Ks ratios (<1.0) for all homologous gene pairs indicate strong purifying selection pressure, suggesting functional conservation despite extensive duplication. This is consistent with the essential role of calcium signaling in fundamental cellular processes and stress responses (Ranty et al., 2006). Interestingly, the Ka/Ks ratios were generally lower for *GmCAM* genes compared to *GmCML* genes, reflecting the higher evolutionary conservation of canonical calmodulins, which are likely to maintain core calcium-sensing functions.

### 4.2 Structural diversity and functional implications

The structural analysis revealed significant diversity in gene architecture and protein features across the GmCAM/CML family. While most *GmCAMs* (10/11) contain introns, a majority of *GmCMLs* (71/102, 69.6%) are intron-less. This pattern is consistent with observations in Arabidopsis and rice (Mccormack et al., 2005; Boonburapong and Buaboocha, 2007), suggesting that the intron-less structure of most CMLs may represent an ancestral state or result from retro position events during evolution. The intron-less nature could potentially allow for more rapid expression in response to stress stimuli, as intron splicing can be rate-limiting during transcription (Jeffares et al., 2008). At the protein level, all identified *GmCAMs* contain four canonical EF-hand domains, consistent with their role as primary calcium sensors. In contrast, *GmCMLs* exhibit greater diversity in the number of EF-hand domains (1-6) and sequence, potentially reflecting functional diversification. The conservation of specific motifs (particularly Motif 1 present in all members and Motif 5 exclusive to *GmCAMs*) suggests distinct functional modules within these proteins. The structural divergence between CAMs and CMLs likely underlies their differential calcium-binding properties and target protein interactions, contributing to the specificity and versatility of calcium signaling responses (Gifford et al., 2007).

The striking structural divergence between *GmCAMs* and *GmCMLs* provides compelling evidence for neofunctionalization following gene duplication events in the soybean CAM/CML gene family (Yang et al., 2024). The contrasting intron-exon organization patterns, with 90.9% of *GmCAMs* retaining introns versus 69.6% of *GmCMLs* being intronless, suggest asymmetric evolutionary trajectories in which duplicated genes acquired distinct functional properties (Glick et al., 2024). The predominant intron-less structure of *GmCMLs* likely represents a key adaptive innovation that enabled specialization for rapid pathogen responses by eliminating time-consuming splicing processes during transcription (Johnson, 2019). This structural streamlining, coupled with diversification in EF-hand domain numbers (1–6 domains in CMLs vs. canonical 4 in CAMs) and sequence divergence in calcium-binding regions, indicates coordinated neofunctionalization that enabled CML proteins to acquire specialized pathogen-sensing capabilities (Zhao et al., 2013). The evolutionary success of this neofunctionalization is demonstrated by the massive expansion of the CML subfamily (102 genes) compared to the conserved CAM subfamily (11 genes), suggesting that the acquisition of pathogen defense functions provided significant adaptive advantages. The identification of 15 *GmCML* genes responding to both viral and fungal pathogens further support the hypothesis that neofunctionalization enabled the evolution of broad-spectrum pathogen recognition capabilities, representing a classic example of how gene duplication followed by functional divergence can generate evolutionary novelty in plant defense systems.

### 4.3 Transcriptional regulation and stress responsiveness

The analysis of cis-acting elements in promoter regions revealed extensive enrichment of hormone-responsive elements, particularly those associated with abscisic acid (31.2%) and methyl jasmonate (27.7%) responses. This suggests that *GmCAM/CML* genes are integrated into hormone signaling networks mediating stress responses, consistent with previous reports in Arabidopsis and rice (Poovaiah et al., 2013). The significant presence of defense and stress-responsive elements (6.5%) and salicylic acid-responsive elements (6.3%) further supports their involvement in biotic stress responses. Interestingly, the dual pathogen-responsive genes identified in our study showed significant enrichment of specific cis-elements compared to the overall family, particularly W-box elements (WRKY binding sites) and TCA elements (salicylic acid-responsive). This enrichment suggests that these genes may be directly regulated by defense-related transcription factors such as WRKYs, which are known to play central roles in plant immunity (Pandey and Somssich, 2009; Wang et al., 2024). The co-occurrence of multiple hormone-responsive elements in these promoters indicates complex hormonal regulation, potentially involving crosstalk between salicylic acid, jasmonic acid, and abscisic acid pathways during pathogen defense.

### 4.4 CAM/CML genes in soybean dual resistance to SMV and *C. sojina*

A major finding of our study is the identification of 19 *GmCAM*/*CML* genes responsive to both SMV and *C. sojina* infection, suggesting their involvement in a common calcium-dependent defense mechanism. This is particularly noteworthy given the distinct nature of these pathogens—SMV is an RNA virus that propagates through the phloem, while *C. sojina* is a necrotrophic fungal pathogen that directly penetrates and kills host cells. The identification of calcium signaling components responsive to both pathogens suggest a role in broad-spectrum disease resistance. Among these dual-responsive genes, *GmCAM4*, *GmCML23*, and *GmCML47* showed the strongest correlation with resistance phenotypes across multiple varieties. *GmCAM4* has been previously implicated in soybean defense responses (Park et al., 2004), but our study is the first to demonstrate its role in resistance to both viral and fungal pathogens. The early and transient expression pattern of *GmCAM4* suggests its involvement in initial signal perception and transmission, consistent with the role of canonical calmodulins in rapid calcium signal decoding (Ranty et al., 2016). The consistent upregulation of *GmCML23* and *GmCML47* in resistant varieties in response to both pathogens, coupled with their strong correlation with resistance phenotypes, indicates their potential roles as key regulators in soybean immune responses. The sustained expression of *GmCML23* throughout the infection time course suggests its involvement in longer-term defense responses, possibly including transcriptional reprogramming and metabolic adjustments. In contrast, the intermediate expression pattern of *GmCML47* may reflect its role in the transition from early to late defense responses. The differential expression patterns of these genes between resistant and susceptible varieties could be attributed to several factors. First, resistant varieties may possess specific allelic variants of these genes that confer enhanced expression in response to pathogen infection. Second, regulatory networks controlling the expression of these genes may differ between resistant and susceptible varieties, possibly due to variations in transcription factor activity or chromatin accessibility. Third, the timing and magnitude of calcium signatures induced by pathogen perception may vary between resistant and susceptible varieties, affecting the downstream activation of CAM/CML genes. The potential regulatory relationship among *GmCAM4*, *GmCML23*, and *GmCML47*, as suggested by their sequential activation patterns, warrants further investigation. *GmCAM4*, being the earliest responder, might directly or indirectly regulate the expression of *GmCML47* and *GmCML23*. Alternatively, these genes might respond to different temporal aspects of calcium signatures induced during pathogen infection.

While we observed that several CAM/CML genes exhibited transcriptional responses to both pathogens, these findings should be interpreted with caution. The apparent similarities or differences in gene expression patterns between the two pathogen treatments cannot be quantitatively assessed due to the lack of standardized experimental controls and uniform methodological approaches. Therefore, any comparative conclusions regarding differential CAM/CML gene regulation in response to SMV versus *C. sojina* infection remain speculative and require validation through future studies employing consistent experimental designs and standardized conditions for both pathogen treatments.

## 5 Conclusion

This study provides the first comprehensive characterization of the CAM/CML gene family in soybean and its potential roles in disease resistance. We identified 113 CAM/CML genes (11 *GmCAMs* and 102 *GmCMLs*) and classified them into 14 distinct phylogenetic groups. Through an integrated analysis of gene structure, protein motifs, chromosomal distribution, and expression patterns, we provide insights into the evolutionary history and functional diversification of this gene family. Our expression analyses in response to two major soybean pathogens, Soybean Mosaic Virus and *C. sojina*, revealed distinct expression patterns of CAM/CML genes associated with resistance. Notably, we identified 19 genes responsive to both pathogens, suggesting their involvement in a shared calcium-dependent defense mechanism. Among these, *GmCAM4*, *GmCML23*, and *GmCML47* showed the strongest correlation with resistance phenotypes, indicating their potential as key regulators of soybean immune responses. In conclusion, our findings enhance our understanding of calcium signaling in soybean disease resistance and identify promising targets for molecular breeding and genetic engineering approaches aimed at developing soybean varieties with enhanced resistance to multiple pathogens. While this study provides valuable insights into the structure and expression patterns of soybean CaM/CML genes, further functional studies including gene knockout, overexpression, and complementation analyses are needed to validate the specific roles of individual CaM/CML genes in pathogen resistance. Such functional validation will be crucial for translating these findings into practical breeding applications for developing disease-resistant soybean cultivars.”

## Data Availability

The datasets presented in this study can be found in online repositories. The names of the repository/repositories and accession number(s) can be found in the article/**Supplementary Material**.

## Supplementary material

Supplementary Figure 1 Heatmap showing expression patterns of all identified CaM/CML genes.
