# Supplementary figures and images for: Comprehensively characterize the soybean CAM/CML gene family, as it provides resistance against both the soybean mosaic virus and Cercospora sojina pathogens
Source: Front Plant Sci. 2025 Jul 21;16:1633325. doi: 10.3389/fpls.2025.1633325 (PMC12318973; doi:10.3389/fpls.2025.1633325)

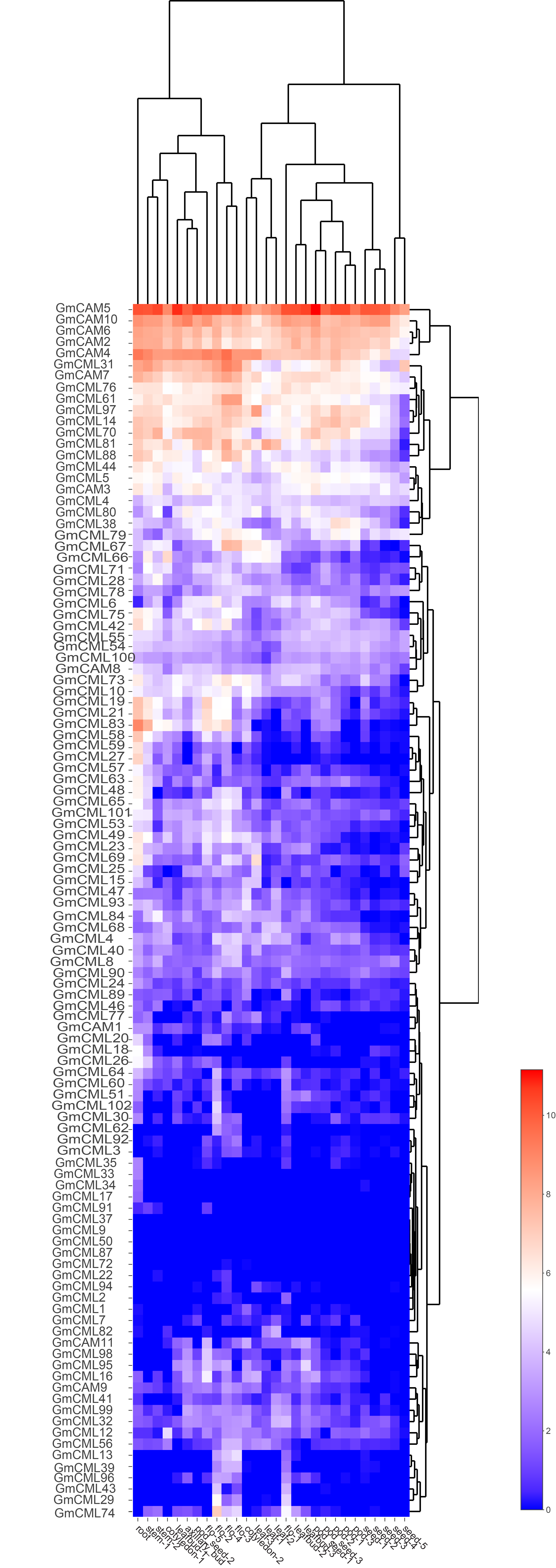

Supplement: Supplementary Figure 1 — Heatmap showing expression patterns of all identified CaM/CML genes. [file Image1.png]
